# Mechanisms involved in hereditary angioedema with normal C1-inhibitor activity

**DOI:** 10.3389/fphys.2023.1146834

**Published:** 2023-05-23

**Authors:** Aleksandr Shamanaev, S. Kent Dickeson, Ivan Ivanov, Maxim Litvak, Mao-Fu Sun, Sunil Kumar, Quifang Cheng, Priyanka Srivastava, Tracey Z. He, David Gailani

**Affiliations:** Department of Pathology, Microbiology and Immunology, Vanderbilt University Medical Center, Nashville, TN, United States

**Keywords:** hereditary angioedema, kallikrein-kinin system, kallikrein, factor XII, plasminogen

## Abstract

Patients with the inherited disorder hereditary angioedema (HAE) suffer from episodes of soft tissue swelling due to excessive bradykinin production. In most cases, dysregulation of the plasma kallikrein-kinin system due to deficiency of plasma C1 inhibitor is the underlying cause. However, at least 10% of HAE patients have normal plasma C1 inhibitor activity levels, indicating their syndrome is the result of other causes. Two mutations in plasma protease zymogens that appear causative for HAE with normal C1 inhibitor activity have been identified in multiple families. Both appear to alter protease activity in a gain-of-function manner. Lysine or arginine substitutions for threonine 309 in factor XII introduces a new protease cleavage site that results in formation of a truncated factor XII protein (Δ-factor XII) that accelerates kallikrein-kinin system activity. A glutamic acid substitution for lysine 311 in the fibrinolytic protein plasminogen creates a consensus binding site for lysine/arginine side chains. The plasmin form of the variant plasminogen cleaves plasma kininogens to release bradykinin directly, bypassing the kallikrein-kinin system. Here we review work on the mechanisms of action of the FXII-Lys/Arg^309^ and Plasminogen-Glu^311^ variants, and discuss the clinical implications of these mechanisms.

## Introduction

The term hereditary angioedema (HAE) encompasses a group of inherited disorders characterized by episodic swelling involving mucosal, submucosal and/or subcutaneous tissues. Edema primarily involves the face, oropharynx, hands, genitals and gastrointestinal tract and may be associated with pain (Kaplan and Joseph, 2010; Schmaier, 2019; Bova et al., 2020; Busse and Christiansen, 2020; Lumry and Settipane, 2020). It is estimated that one in 50,000–100,000 individuals have the condition. Tissue swelling in HAE is usually not associated with itching (urticaria), which helps to distinguish it from more common forms of angioedema induced by histamine release from mast cells (Maurer and Magerl, 2021; Farkas et al., 2022a). Instead, the symptoms of HAE are due primarily to dysregulated formation of the peptide *bradykinin* that promotes vasodilatation and increases vascular permeability (Fields et al., 1983; Kaplan and Joseph, 2010; Schmaier, 2016; De Maat et al., 2018; Schmaier, 2019; Bova et al., 2020; Busse and Christiansen, 2020; Lumry and Settipane, 2020; Wedner, 2020). Bradykinin is normally generated through the activity of the plasma kallikrein-kinin system (KKS).

## The kallikrein-kinin system

**FIGURE 1:**
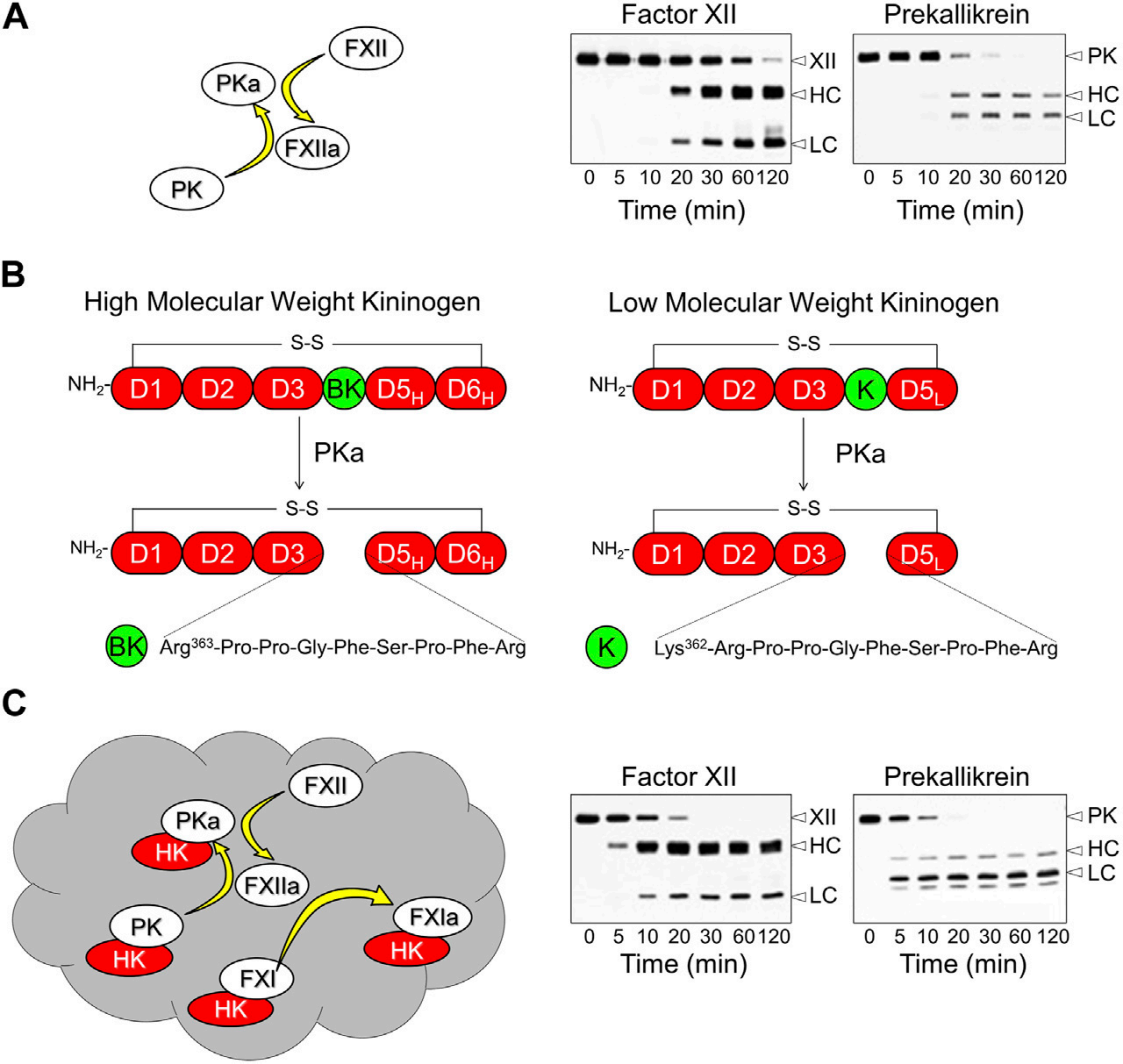
The Kallikrein-Kinin System and Contact Activation. **(A)** In the absence of a surface, factor XII (FXII) and prekallikrein (PK) will reciprocally convert each other to plasma kallikrein (PKa) and factor XIIa (FXIIa) in the absence of a surface. This process involves internal proteolytic cleavage of each protein creating a heavy chain (HC) and catalytic light chain (LC) that remain connected by a disulfide bond. The western blots in the right hand panels of the image are from a time course experiment in which 200 nM FXII and 200 nM PK were incubated together. Blots were run under reducing conditions and were developed with polyclonal antibodies to FXII (left) or PK (right) **(B)** Schematic diagrams of kininogens. High molecular weight kininogen (HK, left) contains six domains (D1-D6) with the bradykinin (BK) sequence within domain 4. The D1-D4 domains of low molecular weight kininogen (LK, right) are identical to those of HK, but the D5 domain is shorter and there is no D6 domain. PKa cleaves HK at two locations to release the nine amino acid bradykinin peptide from HK. It releases BK weakly from LK because of the absence of D6, which contains a binding site for PKa. LK is cleaved preferentially by tissue kallikreins releasing the ten amino acid kallidin (K) peptide. Kallidin is also called lysyl-bradykinin. **(C)** Contact activation on a negatively charged surface (gray cloud) involves autoactivation of FXII, subsequent reciprocal activation of FXII and PK, and FXIIa activation of factor XI (FXI). HK facilitates PK and FXI binding to the surface. Note in the western blots showing FXII and PK cleavage on a contact surface that the reaction proceeds faster than in the blots in panel **(A)**. Adapted from Shamanaev A et al. Recent advances in factor XII structure and function. Curr Opin Hematol. 2022; 29:233–243, and used with permission.

The KKS is comprised of three blood plasma proteins that are synthesized in the liver. Prekallikrein (PK) and factor XII (FXII) are zymogens of the trypsin-like proteases plasma kallikrein (PKa) and FXIIa, respectively (Long et al., 2016; Schmaier, 2016; Srivastava and Gailani, 2020; Girolami et al., 2021). Conversion of PK to PKa, and FXII to FXIIa, involves internal proteolytic cleavage in both zymogens (Figure 1A). As PK is a substrate for FXIIa, and FXII a substrate for PKa, PK, and FXII reciprocally activate each other when mixed in solution (Figure 1A) (Ivanov et al., 2017; Ivanov et al., 2019; Srivastava and Gailani, 2020). Low levels of proteolytic activity intrinsic to zymogen FXII and PK may sustain reciprocal activation in plasma (Ivanov et al., 2017; Ivanov et al., 2020). The third KKS component, high-molecular-weight kininogen (HK), is a 110-kDa glycoprotein that contains the nine amino acid bradykinin sequence within its D4 domain (Figure 1B) (Ponczek, 2021; Dickeson et al., 2022; Kaplan et al., 2022). PKa cleaves HK after Lys^362^ and Arg^371^, releasing the nine amino acid bradykinin peptide. The physiologic and pathologic effects of bradykinin are mediated primarily through the G-protein-coupled B2 receptor, which is constitutively expressed on cell surfaces in many tissues (Lau et al., 2020; Marceau et al., 2020).

Reciprocal activation of PK and FXII is enhanced by a process called contact activation, which occurs when KKS components bind to certain macromolecules or surfaces (Figure 1C) (Colman and Schmaier, 1997; Long et al., 2016; Schmaier, 2016; Petersen et al., 2022). A variety of organic (e.g., nucleic acids, glycosaminoglycans) and inorganic (e.g., polyphosphates, silicates) substances support plasma contact activation (Colman and Schmaier, 1997; Long et al., 2016; Schmaier, 2016; Tillman and Gailani, 2018). Most have a negative surface charge. During contact activation, FXII is autocatalytically converted to FXIIa, initiating more rapid reciprocal activation with PK than occurs in solution (Silverberg et al., 1980a; Long et al., 2016; Girolami et al., 2021). Most PK in plasma circulates as a complex with HK (Mandle et al., 1976; Thompson et al., 1979). HK, in addition to serving as a substrate for PKa, is a cofactor that facilitate PK surface-binding, and may alter PK conformation to make it a better substrate for FXIIa (Wiggins et al., 1977).

The main KKS regulator in plasma is the serpin C1-inhibitor (C1-INH) (Farkas et al., 2022b; Karnaukhova, 2022), encoded by the *SERPING1* gene. C1-INH inhibits PKa and FXIIa, placing a limit on the rate of basal reciprocal turnover of PK and FXII in plasma. This likely maintains bradykinin production within a physiologic range that contributes to setting normal vascular tone and permeability (Iwaki and Castellino, 2006; Revenko et al., 2011). The vascular endothelium likely supports basal bradykinin generation. The components of the KKS assemble on multi-component receptors on vascular endothelial cells comprised of the urokinase plasminogen activator receptor, cytokeratin 1 and the gC1q receptor (Schmaier et al., 1988; Joseph et al., 1999). Binding of KKS proteins to a contact surface at least partially overcomes the capacity of C1-INH to restrict PK and FXII activation, increasing bradykinin production. Local surface-induced contact activation may generate bradykinin at injury sites, promoting vascular leak, tissue edema and pain sensation. It is widely assumed that surface-mediated enhancement of KKS activity is important in HAE, although evidence to support this hypothesis is relatively meager, and the nature of the surfaces involved are not certain.

## Human kininogens

HK is encoded by the *Kng1* gene. The protein is organized into 6 domains (D1 through D6, Figure 1B, left). D4 contains the bradykinin sequence, D5 is involved in surface-binding, and D6 contains a binding site for PK (Silverberg et al., 1980b; Colman and Schmaier, 1997; Ponczek, 2021; Dickeson et al., 2022; Kaplan et al., 2022). *Kng1* also encodes an alternatively spliced mRNA for the plasma protein low-molecular-weight kininogen (LK, Figure 1B, right) (Müller-Esterl et al., 1982; Maier et al., 1983; Sueiras-Diaz et al., 1994; Dickeson et al., 2022; Kaplan et al., 2022). The D1 through D4 domains of LK are identical to those of HK, but D5 is shorter and there is no D6. Because of this, LK interacts weakly with surfaces and with PK and PKa. Consequently, LK is a poor substrate for PKa (Müller-Esterl et al., 1982; Maier et al., 1983; Sueiras-Diaz et al., 1994; Ivanov et al., 2020), and is thought to be primarily a substrate for tissue kallikreins, which cleave it after Met^361^ and Arg^371^ to release the decapeptide lysyl-bradykinin (also called kallidin, Figure 1B), another potent B2 receptor agonist (Lau et al., 2020; Marceau et al., 2020). For our discussion, it is important to recognize that LK is present in plasma at up to four times the concentration of HK (2.4 versus 0.6 μM, respectively) and is, therefore, a greater potential source for vasoactive kinins than is HK.

## Causes of hereditary angioedema

Hypothetically, bradykinin-triggered angioedema could be caused by inherited or acquired conditions that increase kinin formation, increase kinin half-lives, or increase tissue sensitivity to kinins (Table 1, left column). Most patients with HAE have reduced plasma C1-INH activity (5%–30% of normal) due to reduced C1-INH protein (type 1) or a dysfunctional C1-INH variant (type 2) (Table 1, right column) (Kaplan and Joseph, 2010; Schmaier, 2019; Bova et al., 2020; Busse and Christiansen, 2020; Lumry and Settipane, 2020; Farkas et al., 2022b). In these patients, angioedema typically responds to C1-INH infusion (Kaplan and Joseph, 2010; Schmaier, 2019; Bova et al., 2020; Busse and Christiansen, 2020; Lumry and Settipane, 2020; Valerieva and Longhurst, 2022), or drugs that neutralize PK/PKa or FXII/FXIIa (Cohn et al., 2020; Davoine et al., 2020; Busse and Kaplan, 2022), consistent with KKS hyperactivity as an underlying cause. Patients with physical findings and histories consistent with HAE, but with normal C1-INH levels (HAEnC1), were first reported in the year 2000, and may represent 10% or more of all HAE patients (Bork et al., 2000; Santacroce et al., 2021). Six mutations in genes unrelated to C1-INH have been identified in HAEnC1 patients (Table 1, right column) that are assumed to be causative for, or contributory to, angioedema (Dewald and Bork, 2006; Bork et al., 2015; Bafunno et al., 2018; Dewald, 2018; Bork et al., 2019; Ariano et al., 2020; Bork et al., 2021; Veronez et al., 2021). Here we discuss work on mechanisms by which two mutations, one in FXII that changes Thr^309^ to lysine or arginine (Ivanov et al., 2019) and one in plasminogen changing Lys^311^ to glutamic acid (Dickeson et al., 2022), contribute to HAE.

**TABLE 1:** Mechanisms underlying hereditary angioedema.

Possible causes of kinin-induced angioedema	Known inherited causes of kinin-induced angioedema
Increased kinin production	HAE with reduced Cl-INH activity
Reduced C1-INH activity	Reduced C1-INH antigen (type I)
Changes to PK or FXII that enhance reciprocal activation	Reduced C1-INH activity with normal antigen (type 2)
Introduction of surface that enhances PK-FXII reciprocal activation (e.g., over-sulfated chondroitin sulfate)	HAE with normal C1-INH level: Mutations in Secreted Proteins
Novel protease activators of PK or FXII.	Factor XII (Thr328 to Lys or Arg and other exon 9 mutations) Thr309 in this manuscript
Therapeutic activators of PK or FXII that enhance reciprocal activation (e.g., tPA through plasmin)	Plasminogen (Lys33° to Glu) Lys3″ in this manuscript
Novel proteases that cleave kininogens	Angiopoietin-1 (Ala119Ser, Ala8Val or GIn370His)
Changes to kininogens that make them better substrates	Kininogen (Met379Lys)
Increased kinin half-life	HAE with normal Cl-INH—Mutations in Cellular Proteins
Defects in bradykinin degradative pathways	Myoferlin Arg217 To Ser—may enhance EGF signaling
Therapeutic inhibitors of kinin degradation (e.g., ACE inhibitors)	Heparan sulfate (HS)-glucosamine 3-O[sulfotransferase 6] (Thr144 to Ser) may interfere with protein glycosylation
Increased tissue sensitivity to kinins	
Increased signaling through bradykinin receptors	

## Factor XII

**FIGURE 2:**
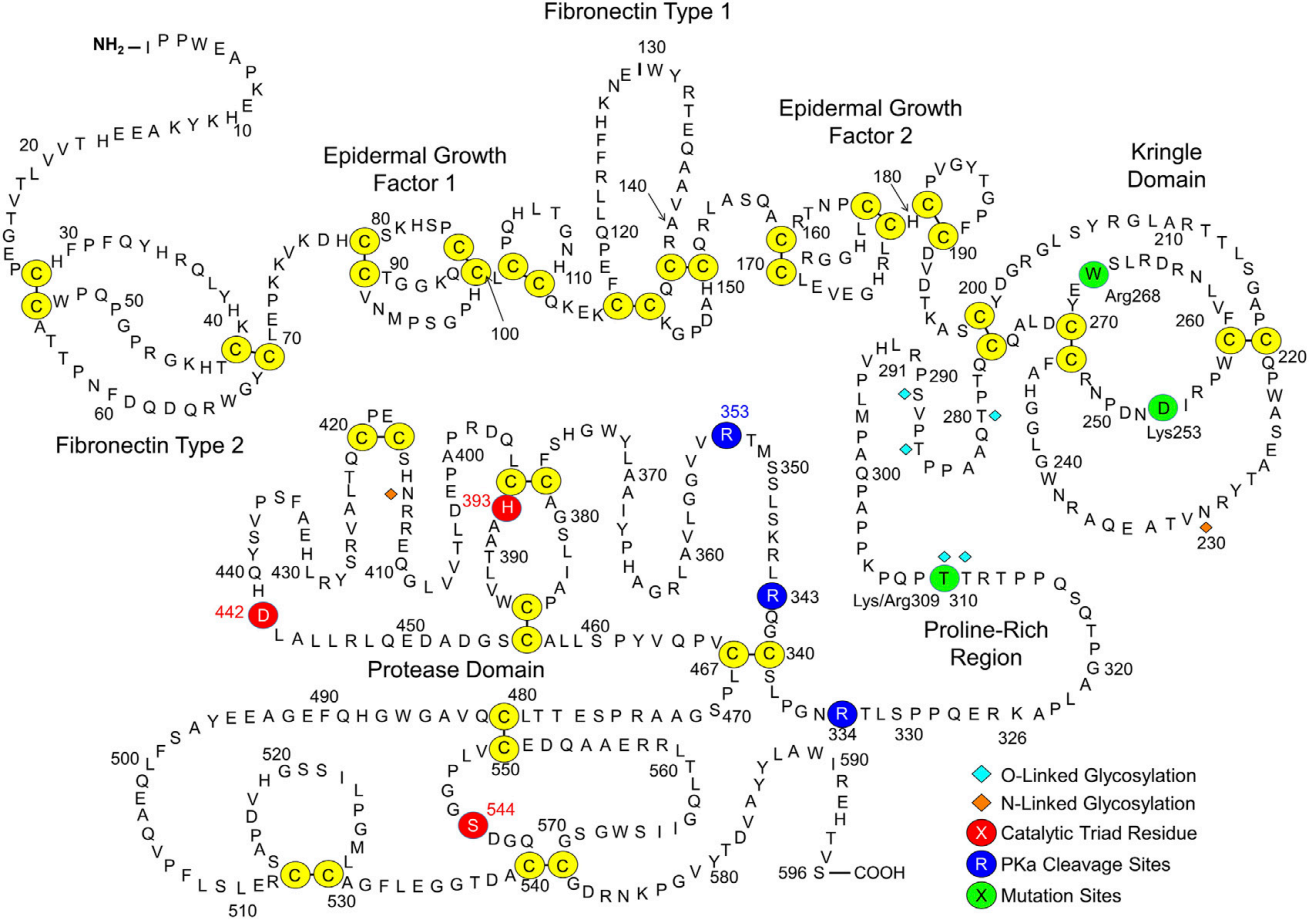
Factor XII. Shown is the amino acid sequence and domain structure of human plasma FXII. The FXII heavy chain contains fibronectin type 2, epidermal growth factor 1, fibronectin type 1, epidermal growth factor 2 and kringle domains, and a proline-rich region. The protease domain is a trypsin-like catalytic unit. Disulfide bonds between cysteine residues are shown in yellow. The catalytic triad (His^393^, Asp^442^, and Ser^544^) is indicated by red circles. Cleavage sites for PKa (Arg^334^, Arg^343^, Arg^353^) are indicated by dark blue circles. Adapted from Shamanaev A et al. Recent advances in factor XII structure and function. Curr Opin Hematol. 2022; 29:233–243, and used with permission.

FXII is an 80-kDa polypeptide encoded by the *F12* gene that is synthesized primarily in hepatocytes (Cool et al., 1985; de Maat and Maas, 2016; Maas and Renné, 2018; Shamanaev et al., 2022a; Shamanaev et al., 2022b). Figure 2 shows the amino acid sequence and predicted domain structures for human FXII (Cool et al., 1985; Shamanaev et al., 2022a; Shamanaev et al., 2022b). The N-terminal isoleucine of the protein in plasma is designated residue 1. From the N-terminus, FXII contains a fibronectin type 2 (FN2), first epidermal growth factor (EGF1), fibronectin type 1 (FN1), second epidermal growth factor (EGF2), and kringle (KNG) domain, a proline-rich region (PRR), and a trypsin-like protease domain. Conversion of FXII to FXIIa requires proteolytic cleavage after Arg^353^, creating a heavy chain (amino acids 1–353) and light chain (amino acids 354–596) that remain connected by the Cys^340^-Cys^467^ disulfide bond. The heavy chain has several functions. First, when FXII is in solution (not bound to a surface), elements of the heavy chain maintain the protein in a “closed” conformation that is relatively resistant to activation by PKa (de Maat et al., 2019; Ivanov et al., 2019; Clark et al., 2020; Shamanaev et al., 2022a; Shamanaev et al., 2022b). Second, during contact activation, the heavy chain mediates FXII surface-binding, opening the protein conformation to facilitate activation (de Maat et al., 2019; Shamanaev et al., 2022a; Shamanaev et al., 2022b). Third, once FXII is converted to FXIIa, the heavy chain keeps the protease associated with the surface (de Maat and Maas, 2016; Maas and Renné, 2018; Shamanaev et al., 2022a; Shamanaev et al., 2022b), where it efficiently activates FXII (autoactivation) and PK.

## HAE caused by factor XII Lys/Arg substitutions for threonine 309

**FIGURE 3:**
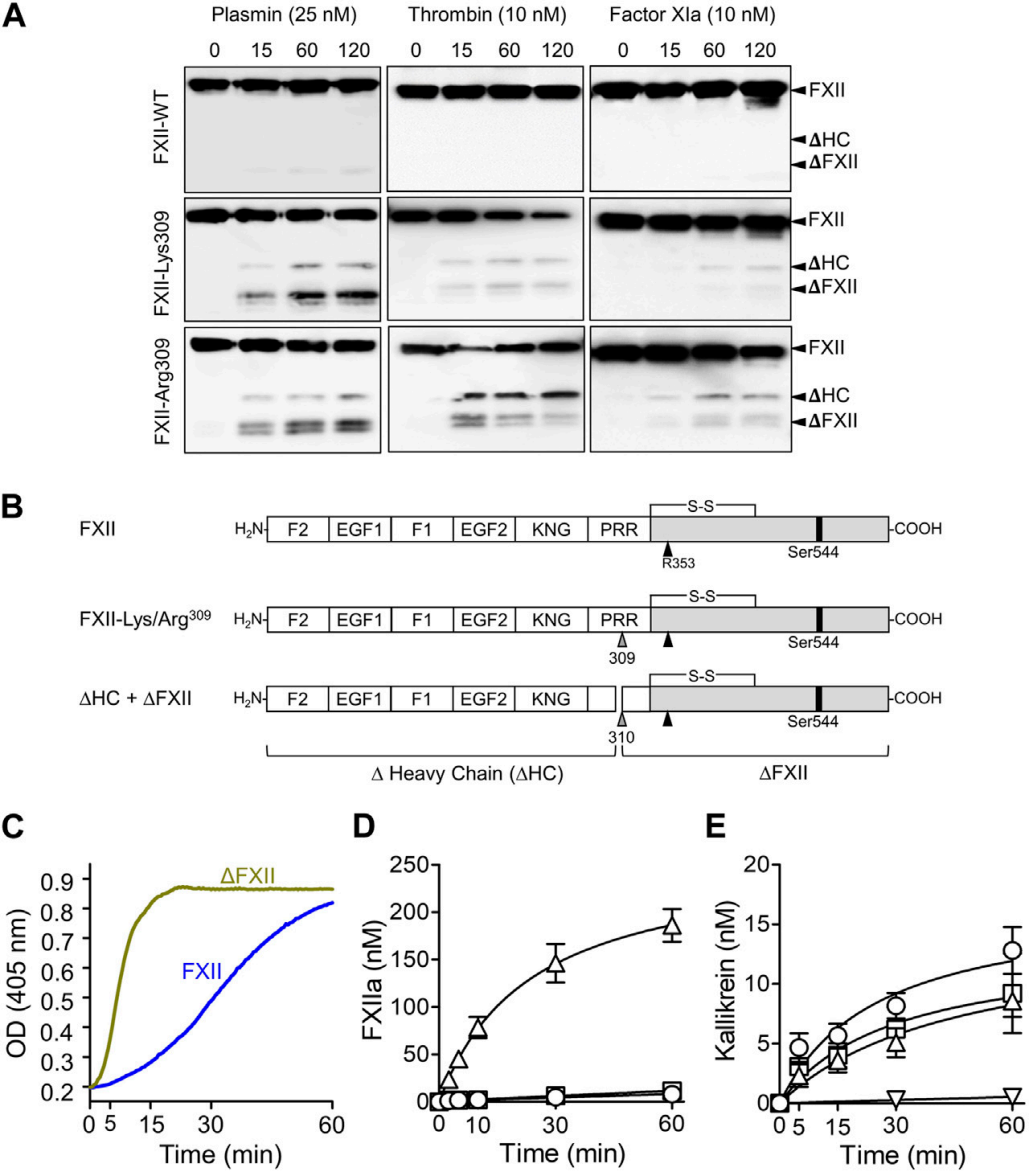
ΔFXII. **(A)** Non-reducing Western blots for time courses of FXII species (200 nM) cleavage by 25 nM plasmin (left column), 10 nM thrombin (center) or 10 nM factor XIa. Wild type FXII (top row), FXII-Lys^309^ (middle row), FXII-Lys^309^ (bottom row). Blots were developed with a mixture of monoclonal IgGs to the FXII heavy and light chains. Positions of standards for FXII (FXII), the heavy (HC) and light (LC) chains of FXIIa, the heavy chain of FXII-Lys^309^ or FXII-Arg^309^ cleaved after residue 309 (ΔHC) and FXII residues Thr^310^ to Ser^596^ (**Δ**FXII). **(B)** Schematic diagrams of wild type FXII and FXII with lysine or arginine replacement of Thr^309^ (gray arrow). Cleavage after Lys/Arg^309^ creates two proteins, **Δ**-heavy chain (**Δ**HC) and **Δ**FXII. **(C)** FXII-PK Reciprocal activation. PK (60 nM) was mixed with 12.5 nM FXII or ∆FXII, and 200 nM chromogenic substrate S-2302 at 37°C. Changes in optical density (OD) at 405 nm were continuously monitored. The signals are created by the activities of kallikrein and FXIIa. **(D)** Activation of 200 nM FXII (O), the full-length FXII precursor used to generate ∆FXII (•), or ∆FXII (△) by PKa (10 nM) at 37°C. FXIIa activity was measured by chromogenic assay. **(E)** PK (60 nM) was incubated with 50 pM FXIIa (O), βFXIIa (□) or **Δ**FXIIa-3C (△), or no FXIIa (▽) at 37°C. At various times PKa generation was determined by chromogenic assay. **(A–D)** are from Ivanov et al. (2019), and **(C)** is from Shamanaev et al. (2022a).

In 2006, Dewald and Bork described single base pair changes in exon 9 of the *F12* gene in some HAEnC1 patients (Dewald and Bork, 2006). The mutations result in replacement of Thr^309^ in the PRR (Thr^328^ if counting from the initiator methionine on the signal peptide) with either lysine or arginine (Figure 2). The substitution disrupts an N-linked glycosylation site at residue 309 (Björkqvist et al., 2015) and may affect an adjacent glycosylation site at Thr^310^. Lys/Arg^309^ and a few rarer F12 exon 9 mutations have been identified in more than 150 families with HAE (Dewald and Bork, 2006; Bork et al., 2015; Santacroce et al., 2021; Veronez et al., 2021). Introducing a basic amino acid into a protein sequence may create a novel cleavage site for trypsin-like proteases. In 2016 de Maat et al. showed that the fibrinolytic protease plasmin cleaves FXII-Lys/Arg^309^ variants after residue 309 (Figure 3A) (de Maat et al., 2016). Subsequently, we reported that the coagulation proteases thrombin and factor XIa cleave FXII-Lys/Arg^309^ at the same site (Figure 3A) (Ivanov et al., 2019), perhaps explaining why patients with Lys/Arg^309^ substitutions often have attacks of angioedema following trauma that activates the coagulation mechanism (Müller-Esterl et al., 1982). Cleavage after Lys/Arg^309^ separates most of the heavy chain from the protease domain, resulting in a truncated FXII (ΔFXII, Figure 3B) that cannot bind properly to surfaces (Ivanov et al., 2019).

## ΔFXII and the kallikrein-kinin system

As shown in Figure 1, mixing PK and FXII in solution leads to reciprocal activation of both proteases, and the rates of both reactions are increased when a surface is added (Srivastava and Gailani, 2020; Shamanaev et al., 2022a; Shamanaev et al., 2022b). Replacing FXII with ΔFXII accelerates reciprocal activation with PK in the absence of a surface (Figure 3C) (Ivanov et al., 2019; Shamanaev et al., 2022a; Shamanaev et al., 2022b). Indeed, ΔFXII accelerates reciprocal activation with PK to a degree similar to FXII variants that lack heavy chain regulatory function (Shamanaev et al., 2022a; Shamanaev et al., 2022b). This suggests that ΔFXII is activated faster than FXII by PKa, that PK is activated more rapidly by ΔFXIIa than by FXIIa, or a combination of the processes. ΔFXII is activated by PKa at least 15-fold more rapidly than is FXII (Figure 3D) (Ivanov et al., 2019), consistent with absence of heavy chain regulatory function that normally maintains FXII in a closed conformation (Ivanov et al., 2019; Shamanaev et al., 2022a). In contrast, ΔFXIIa and full-length FXIIa activate PK at roughly similar rates, indicating the heavy chain is not required for PK activation in the absence of a surface (Figure 3E) (Ivanov et al., 2019). Interestingly, adding surface to reactions containing ΔFXII and PK does not accelerate activation as in reactions with full-length FXII, probably because FXII requires its heavy chain to bind to a surface (Ivanov et al., 2019).

**FIGURE 4:**
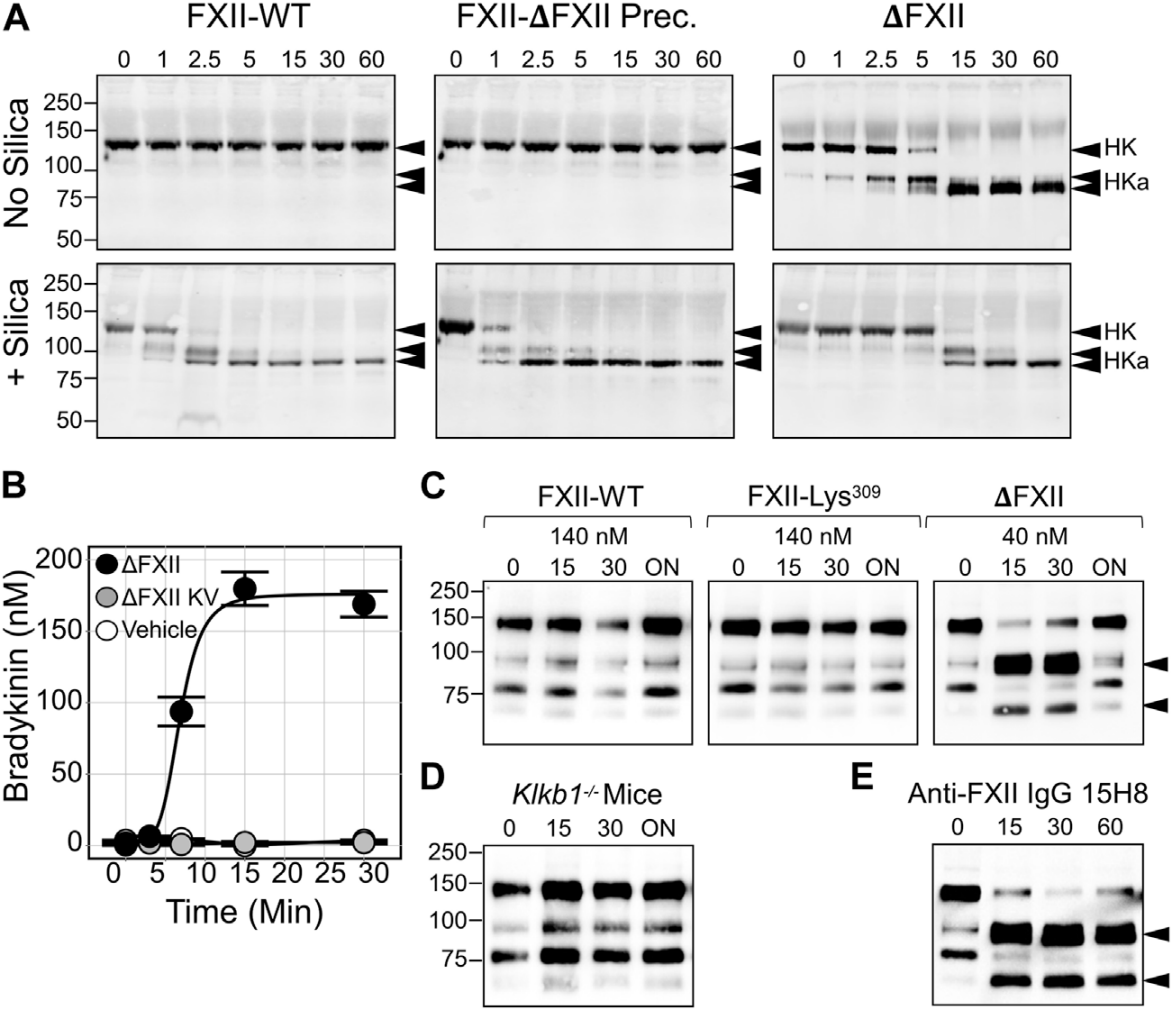
Effects of ΔFXII on the KKS. **(A)** HK cleavage in human plasma. Shown are western blots of human FXII-deficient plasma supplemented with FXII-WT. The full-length precursor of ∆FXII or ∆FXII (400 nM) in the absence (−) or presence (+) of a silica-based reagent that induces contact activation. At indicated times samples were removed into non-reducing sample buffer. Western blots were probed with goat anti-human HK IgG (HK). Positions of standards for HK, and the two bands of cleaved HK (HKa), are shown on the right. Positions of molecular mass standards in kilodaltons are shown to the left of the images. **(B)** Bradykinin generation in normal plasma after addition of 160 nM ∆FXII (•), ∆FXII and the PKa inhibitor (KV999272 10 nM; •), or vehicle (O). Bradykinin was measured by ELISA. **(C)** FXII-WT, FXII-Lys^309^ or ∆FXII were administered intravenously to wild-type C57Bl/6 mice to an estimated final plasma concentration of 140 nM or 40 nM. Shown are non-reducing western blots of plasma collected 0, 15, 30 min, or ∼18 h (ON, for overnight) after FXII infusion. Blots were developed with anti-murine HK IgG (anti-mHK). **(D)** As in **(C)**, except that 40 nM ∆FXII was infused into Klkb1 null (PK deficient mice). **(E)** As in **(C)**, except that FXII-deficient mice replete with 140 nM FXII-WT were treated with monoclonal antibody 15H8, which binds to the human FXII FN2 domain. For **(C,E)**, the positions of bands indicating cleaved HK are indicated by black arrows. **(A–E)** are from Ivanov et al. (2019). **(B)** is from Dickeson et al. (2022).

These observations support a model in which FXII truncation results in a protein that is activated at a supraphysiologic rate by PKa. The greater amount of activated FXIIa protease generated (ΔFXIIa, in this case), in turn, accelerates PK activation. Accelerated reciprocal activation mediated by ΔFXII may overwhelm the capacity of C1-INH at physiologic concentrations to control the reaction, resulting in a surface-independent increase in bradykinin. Consistent with this hypothesis, adding ΔFXII, but not full-length FXII or the full-length precursor of ΔFXII (FXII-Lys/Arg^309^), to human plasma results in rapid HK cleavage (Figure 4A, top row) and bradykinin release (Figure 4B) (Ivanov et al., 2019). Running the plasma reactions in the presence of a surface (silica) accelerates HK cleavage with full-length FXII and FXII-Lys/Arg^309^, but does not change the effect of ΔFXII (Figure 4A, bottom row), which cannot bind to the surface. HK cleavage occurs rapidly after intravenous infusion of ΔFXII into wild type C57Bl/6 mice, but does not occur after infusion of FXII or FXII-Lys/Arg^309^ (Figure 4C). HK cleavage does not occur after infusing ΔFXII into mice lacking PK (Figure 4D, left panel), and ΔFXII does not induce bradykinin generation in normal human plasma that contains the potent PKa inhibitor KV999272 (Figure 4B), supporting the hypothesis that ΔFXII causes marked acceleration of the plasma KKS.

## ΔFXII—clinical and therapeutic implications

Angioedema caused by ΔFXII may be one example of a broader phenomenon involving loss of intrinsic regulation of FXII activation via removal or disruption of the heavy chain, rather than loss of extrinsic regulation due to C1-INH deficiency. Scheffel *et al.* described a FXII Trp^268^ to arginine substitution in patients with a novel autoinflammatory syndrome (Scheffel et al., 2020). Hofman et al. showed that Arg^268^ disrupts the closed conformation of FXII, facilitating rapid activation and truncation within the KNG domain (Figure 2) (Hofman et al., 2020). Zamolodchikov et al. described an alternatively spliced FXII mRNA expressed in neurons that encodes a truncated FXII (the first amino acid is FXII residue 297) found in cerebrospinal fluid of patients with Alzheimer disease or multiple sclerosis (Figure 2) (Zamolodchikov et al., 2019). Like ΔFXII, this truncated protein is rapidly activated by PKa. de Maat and co-workers reported that truncation of wild type human FXII within the PRR by neutrophil elastase or cathepsin K results in proteins that are rapidly activated by PKa (Figure 2) (de Maat et al., 2019).

Novel inhibitors directed at FXII and FXIIa are being developed to treat or prevent thrombotic disorders (Fredenburgh and Weitz, 2021; Kluge et al., 2022). Because individuals lacking FXII do not have obvious abnormalities related to the deficiency, a long-acting compound such as an antibody could hypothetically be used to safely block FXII activation on a long-term basis. Antibodies to the FXII heavy chain can be used to specifically block surface-induced FXII activation, reducing factor XI activation by FXIIa, and subsequent thrombin generation. Indeed, this approach was effective in a baboon thrombosis model using a monoclonal anti-FXII IgG (15H8) that binds to the FXII FN2 domain (Figure 2) (Matafonov et al., 2014). However, while 15H8 blocked contact activation, it also disrupted the regulatory activity of the heavy chain that limits FXII activation, leading to a surge in PK activation and HK cleavage *in vivo*, comparable to what is observed with ΔFXII infusion (Figure 4E) (Ivanov et al., 2019). Antithrombotic strategies targeting the FXII(a) protease domain would, therefore, seem preferable, because they would not increase surface-independent FXII activation and the associated risk of angioedema.

## Plasminogen, plasmin, and the KKS

**FIGURE 5:**
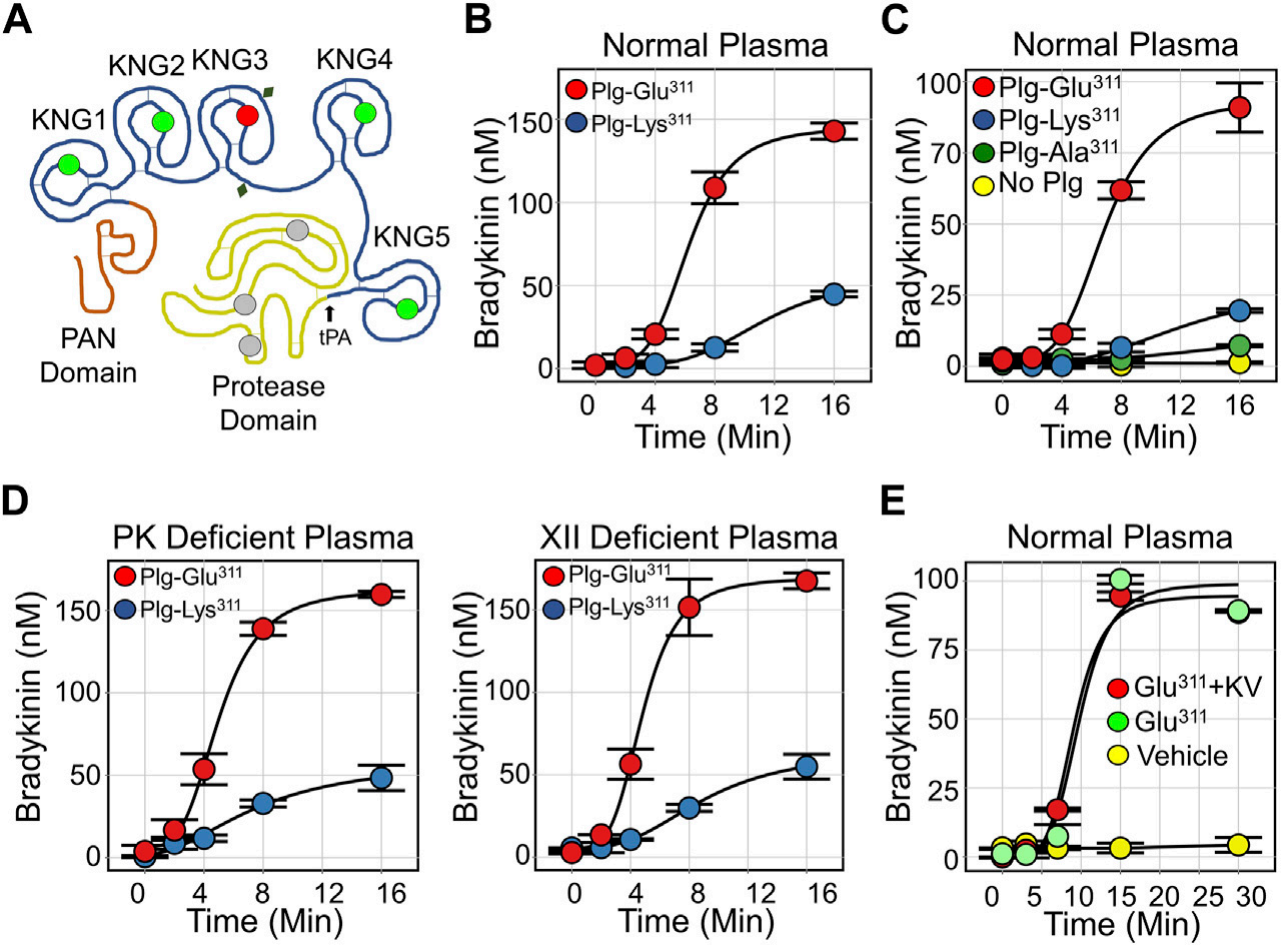
Plasmin and Bradykinin Production. **(A)** Diagram of human plasminogen (PLG), showing the N-terminal PAN domain, five kringle domains (KNG1-5) and the protease domain. Locations of lysine/arginine-binding Asp-X-Asp/Glu motifs are indicated by green circles, and the location of Lys^311^ is indicated by the red circle. The catalytic triad is indicated in gray and glycosylation sites by the diamonds. The site at which tPA cleaves PLG to generate plasmin is indicated by a black arrow. **(B)** Bradykinin generation in normal plasma supplemented with 600 nM (final concentration) Plg-Lys^311^ (blue) or Plg-Glu^311^ (red) after addition of tPA to 50 nM. **(C)** Bradykinin generation in normal plasma supplemented with 600 nM Plg-Lys^311^ (blue), Plg-Ala^311^ (green), Plg-Glu^311^ (red) or vehicle (yellow) after addition of tPA to 50 nM. **(D)** Bradykinin generation in PK-deficient plasma (left) or FXII-deficient (right) plasma supplemented with 600 nM (final concentration) Plg-Lys^311^ (blue) or Plg-Glu^311^ (red) after addition of tPA to 50 nM. **(E)** Bradykinin generation in normal plasma supplemented with 600 nM Plg-Glu^311^ (red, green), or vehicle (yellow) in response to tPA (50 nM) in the absence (green) or presence (red) of 10 μM KV999272. **(B–E)** are from Dickeson et al. (2022).

Plasminogen is the 90 kDa zymogen of the protease plasmin (Novokhatny, 2008; Law et al., 2012; Law et al., 2013). It is encoded by the *PLG* gene. From the N-terminus, full-length human Glu-plasminogen (Figure 5A) contains a PAN domain, five kringle domains (KNG1-KNG-5), and a trypsin-like protease domain. The KNG1, KNG2, KNG4 and KNG5 domains of plasminogen contain Asp-X-Asp/Glu motifs that bind side chains of lysine and arginine residues (Novokhatny, 2008; Law et al., 2012; Law et al., 2013). In Glu-plasminogen, these motifs are involved in intramolecular binding interactions that maintain the zymogen in a closed conformation that is activated slowly by the plasminogen activator tPA. The FXII KNG domain seems to contribute to a closed conformation by a similar mechanism (Shamanaev et al., 2022a; Shamanaev et al., 2022b). Glu-plasminogen binding to fibrin, like FXII binding to a surface, results in conformational changes that expose the activation cleavage site, increasing the rate of activation by tPA (Urano et al., 2018).

Plasmin contributes to multiple processes including degradative reactions, tissue remodeling and inflammation (Maas, 2019; Heissig et al., 2020; Rahman and Krause, 2020; Keragala and Medcalf, 2021). It is a promiscuous protease that cleaves numerous plasma proteins, including the three components of the KKS. Plasmin has long been recognized as a FXII activator (Kaplan and Austen, 1971), although its ability to cleave the protein after Arg^353^ is relatively weak when compared to PKa (Kaplan and Austen, 1971; de Maat et al., 2016; Dickeson et al., 2022). It can also slowly convert PK to PKa (Dickeson et al., 2022). While plasmin readily cleaves HK (Marcos-Contreras et al., 2016; Henderson et al., 2021; Dickeson et al., 2022); however, it is not clear that bradykinin is a major product of the reaction. FXII activation by plasmin appears to be at least 20 to 50-fold slower than with PKa (Joseph et al., 2017; Dickeson et al., 2022). The lysine analog *ε*-aminocaproic acid (Amicar) inhibits plasmin cleavage of HK, indicating that binding interactions between the Asp-X-Asp/Glu motifs on plasmin KNG domains and lysine/arginine residues on HK are involved in the interaction (Kleniewski and Donaldson, 1987; Dickeson et al., 2022).

## HAE caused by a plasminogen glutamic acid substitution for lysine 311

In 2018 Bork et al. and Dewald et al. described a point mutation in the *PLG* genes of two HAEnC1 patients that replaces Glu-plasminogen Lys^311^ with glutamic acid (Lys^330^ if counting from the initiator methionine on the signal peptide) (Bork et al., 2015; Dewald, 2018). The substitution has been found in over 150 individuals from more than 30 families on three continents, suggesting a wide distribution (Bork et al., 2018; Santacroce et al., 2021; Veronez et al., 2021). Lys^311^ is in the KNG3 domain (Figure 5A), the only one of the five plasminogen KNG domains lacking an Asp-X-Asp/Glu motif (Law et al., 2012; Law et al., 2013). It is Lys^311^ that disrupts what would otherwise be an intact Asp-X-Asp/Glu motif (Asp-X-Lys), and the Glu^311^ substitution in HAE patients creates a new lysine/arginine binding site (Asp-X-Glu). This suggests Glu^311^ is a gain-of-function mutation. In collaboration with Ruby Law, James Whisstock and Adam Quek of Monash University, we studied wild type and mutant plasminogens (Plg-Lys^311^ and Plg-Glu^311^, respectively) to determine how Plg-Glu^311^ might cause angioedema (Dickeson et al., 2022).

## Plasminogen Glu^311^ in plasma assays

When tPA is added to normal plasma supplemented with recombinant plasminogen, bradykinin generation is much greater with Plg-Glu^311^ than with Plg-Lys^311^ (Figure 5B) (Ivanov et al., 2020). Activity associated with Glu^311^ cannot be attributed specifically to loss of Lys^311^, as plasminogen with an Ala^311^ substitution did not enhance bradykinin release (Figure 5C). The effect of Plg-Glu^311^ is also observed in plasmas lacking FXII or PK (Figure 5D), indicating that bradykinin is somehow produced independently of the KKS. In studies using purified proteins, Plg-Glu^311^ and Plg-Lys^311^ are activated at comparable rates by tPA. Both zymogens are also weakly activated by PKa and FXIIa (Dickeson et al., 2022). Furthermore, the active plasmin forms (Plm-Glu^311^ and Plm-Lys^311^) both weakly catalyze FXII and PK activation. Taken together, these data suggest the Glu^311^ substitution confers a gain-of-function that facilitates bradykinin release independently of the KKS. Indeed, a potent PKa inhibitor (KV999272) has no effect on kinin formation in tPA-treated plasma containing Plg-Glu^311^ (Figure 5E), while blocking kinin formation induced by addition of ΔFXII (Figure 4B).

## Plasmin Glu^311^ cleavage of human kininogens

**FIGURE 6:**
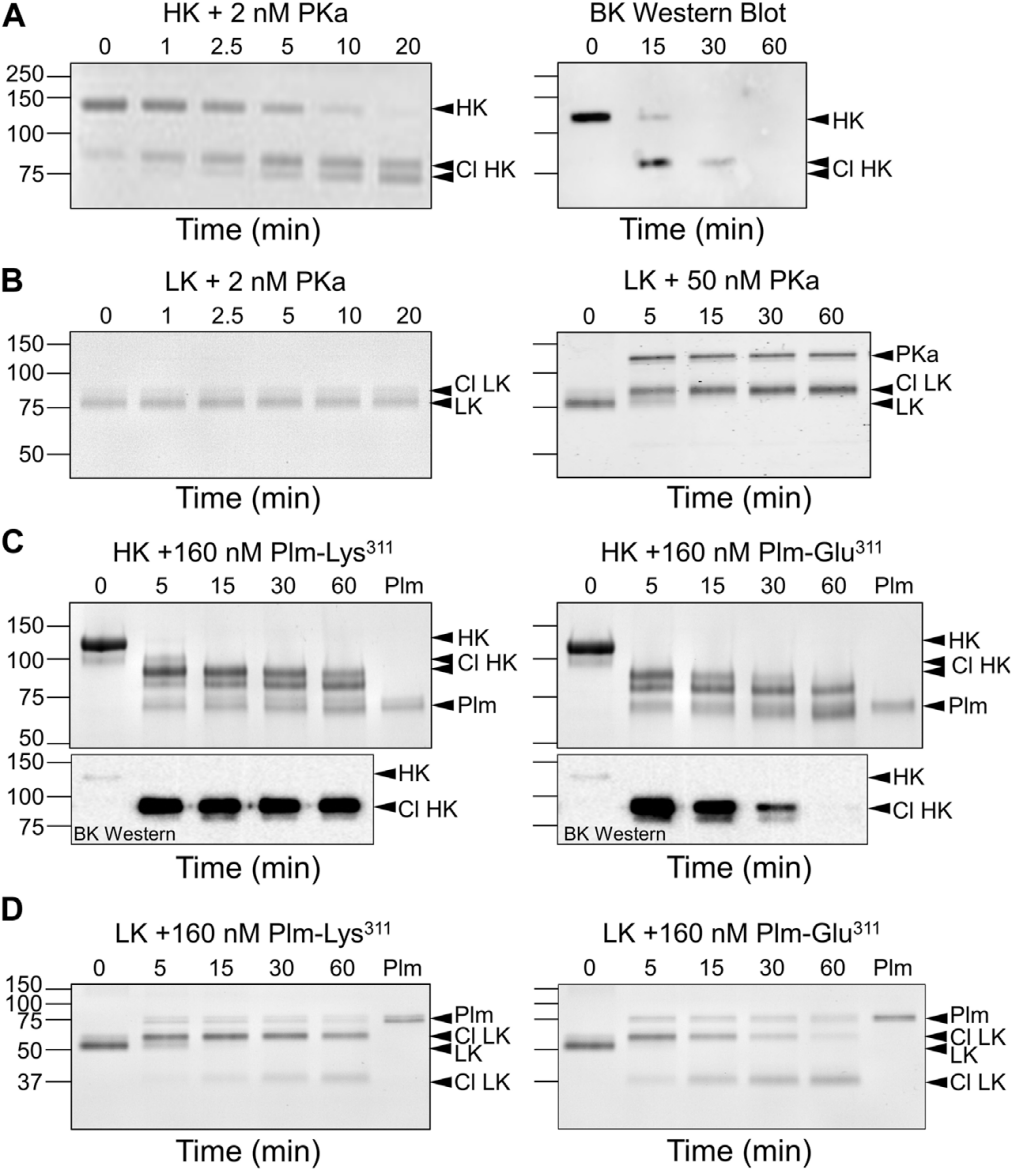
Kininogen Cleavage by Kallikrein and Plasmin. Coomassie Blue-stained SDS-polyacrylamide gels of time courses of PKa **(A,B)** or plasmin **(C,D)** cleavage of HK **(A,C)** or LK **(B,D)**. At indicated times, samples were removed into non-reducing sample buffer, size-fractionated on 10% SDS-PAGE, followed by staining. **(A)** Human plasma-derived HK (200 nM) was incubated with PKa (2 nM) at 37°C. The image on the right is a Western blot of a similar reaction using an antibody to bradykinin. **(B)** Human plasma-derived LK (200 nM) was incubated with 2 nM (left) or 50 nM (right) PKa at 37°C. **(C)** Top Panels*.* Human plasma-derived HK (800 nM) incubated with 160 nM Plm-Lys^311^ (left) or Plm-Glu^311^ (right). Bottom Panels*.* Western blot of samples from reactions similar to those in the Top Panels using an antibody to bradykinin. **(D)** Human plasma-derived LK (200 nM) incubated with 160 nM Plm-Lys^311^ or Plm-Glu^311^. For all panels positions of molecular mass standards in kDa are indicated on the left. Positions of standards for uncleaved HK or LK, cleaved forms of HK (Cl HK) or (Cl LK), and kallikrein (PKa) or plasmin (Plm) are indicated on the right. All images are from Dickeson et al. (2022).

HK and LK contain disulfide bonds (Cys^10^-Cys^596^ and Cys^10^-Cys^389^, respectively) that connect their N- and C-termini (Figure 1B) (Ponczek, 2021; Dickeson et al., 2022; Kaplan et al., 2022). PKa cleavage of the HK Arg^371^-Ser^372^ peptide bond leads to a pronounced shift in HK migration on non-reducing SDS-PAGE (Figure 6A, left panel), consistent with a change in conformation from a circular to more linear extended form (Ivanov et al., 2020). Cleavage of the Lys^362^-Arg^363^ bond then releases bradykinin, causing a second subtler downward shift. Western blots using an anti-bradykinin IgG indicate the BK sequence remains associated with HK after one cleavage, and is released by the second (Figure 6A, right panel). LK cleavage by PKa causes a slight upward shift in migration (Figure 6B, left panel) and relatively high PKa concentrations are required to observe cleavage (Figure 6B, right panel) (Dickeson et al., 2022). While both Plm-Lys^311^ and Plm-Glu^311^ readily cleave HK (Figure 6C, top panels), western blots indicate bradykinin is released by Plm-Glu^311^ more rapidly than by Plm-Lys^311^ (Figure 6C, bottom panels). A similar shift to that observed with PKa occurs when LK is incubated with Plm-Lys^311^ or Plm-Glu^311^ (Figure 6D).

## Plasmin Glu^311^ and kinin generation

**FIGURE 7:**
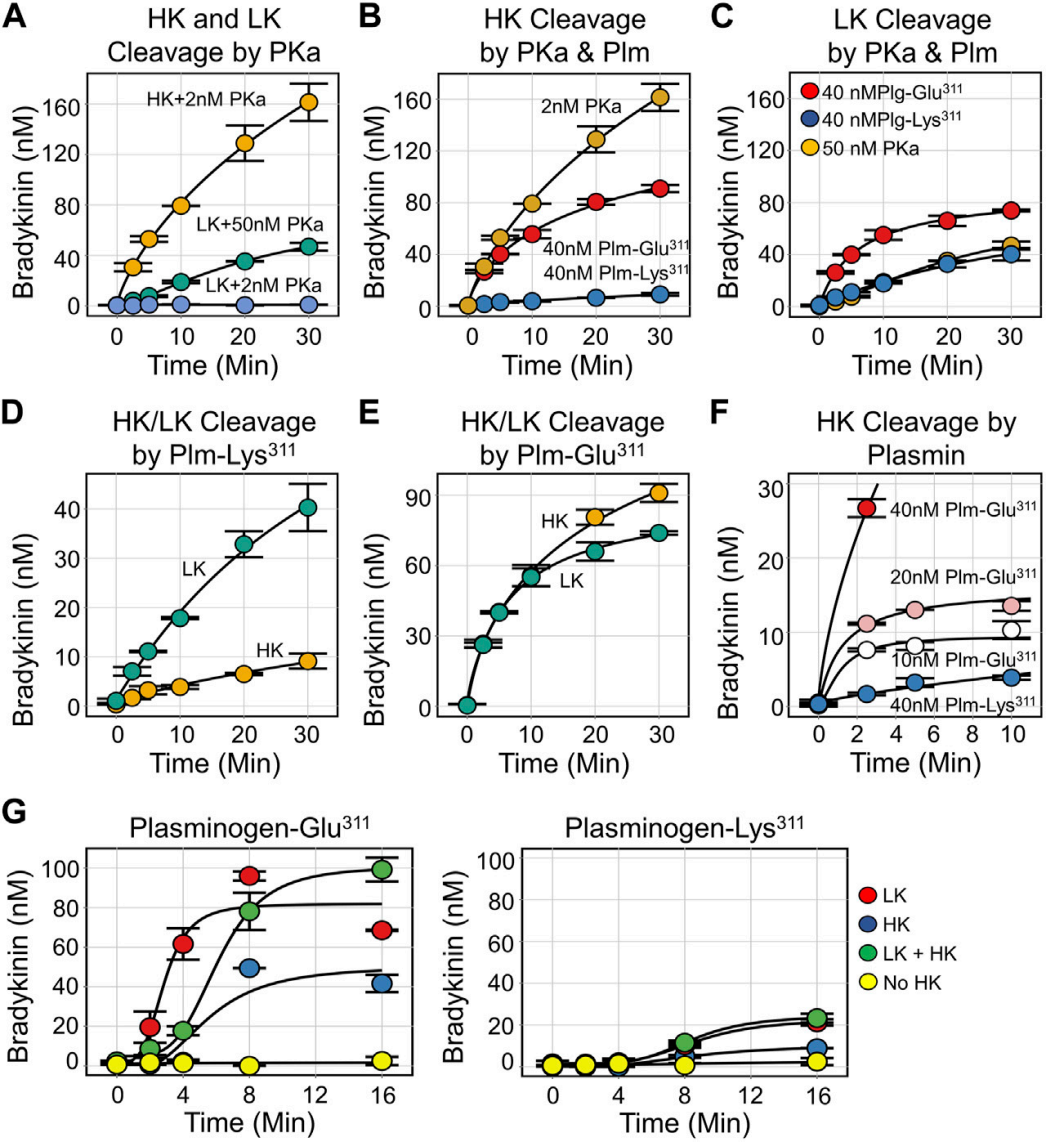
Kinin Generation by Kallikrein and Plasmin. For all reactions, samples were collected at indicated time points and bradykinin concentration determined by ELISA. **(A)** Plasma-derived HK (200 nM) incubated with 2 nM PKa (orange), plasma-derived LK (200 nM) incubated with 50 nM PKa (green) or 2 nM PKa (blue). **(B)** Plasma-derived HK (200 nM) incubated with 2 nM PKa (orange), 40 nM Plm-Glu^311^ (red), or 40 nM Plm-Lys^311^ (blue). **(C)** Plasma-derived LK (200 nM) incubated with 50 nM PKa (orange), 40 nM Plm-Glu^311^ (red), or 40 nM Plm-Lys^311^ (blue). **(D)** Plasma derived HK (orange) or LK (green), 200 nM, incubated with 40 nM Plm-Lys^311^. **(E)** Plasma derived HK (orange) or LK (green), 200 nM, incubated with 40 nM Plm-Glu^311^. **(F)** Plasma-derived HK (200 nM) incubated with 40 nM (red), 20 nM (pink), or 10 nM (white) Plm-Glu^311^ or 40 nM Plg-Lys^311^ (blue). **(G)** Plasma from a patient deficient in HK and LK was supplemented with 600 nM plasma-derived HK (blue), 2.3 μM plasma-derived LK (red), HK, and LK (green) or vehicle (yellow). Plm-Glu^311^ (left) or Plm-Lys^311^ (right) were added to a final concentration of 600 nM, and tPA (50 nM) was added to activate plasminogen. All images are from Dickeson et al. (2022).

We measured kinin production from HK and LK incubated with plasmin using an ELISA that detects bradykinin and lysyl-bradykinin comparably (Dickeson et al., 2022). In all cases, mass spectroscopic analysis confirmed that the released peptide was bradykinin. Consistent with published literature, PKa releases bradykinin from HK 50–100-fold faster than from LK (Figure 7A). Bradykinin release from HK catalyzed by wild type Plm-Lys^311^ is at least 50-fold slower than in reactions with PKa (Figure 7B), while PKa and Plm-Lys^311^ release bradykinin from LK at similar rates (Figure 7C). Interestingly, Plm-Lys^311^ releases bradykinin ∼3-4 fold faster from LK than from HK (Figure 7D). Given that LK is two to four times more abundant in plasma than HK, these findings suggest that kinin release from kininogens catalyzed by wild type plasmin would primarily come from LK.

The initial rates of bradykinin release catalyzed by Plm-Glu^311^ is at least 10-fold faster for HK (Figure 7B), and 2-3-fold faster for LK (Figure 7C), than with Plm-Lys^311^ (Dickeson et al., 2022). Indeed, with Plm-Glu^311^, bradykinin release is comparable for HK and LK (Figure 7E). For HK, peak bradykinin generation roughly correlates with the Plm-Glu^311^ concentration (Figure 7F), suggesting a stochiometric interaction rather than a process with classic Michaelis-Menten kinetics. Plasma from a person deficient in HK and LK was supplemented with physiologic concentrations of HK (640 nM), LK (2.3 μM) or both. Plg-Lys^311^ or Plg-Glu^311^ was added, followed by tPA to generate plasmin (Figure 7G). For both Plg-Lys^311^ and Plg-Glu^311^, bradykinin generation was greater in plasma containing LK than in plasma containing HK, consistent with LK being the main target for plasmin. Bradykinin generation was substantially greater with Plg-Glu^311^ than Plg-Lys^311^.

## Plasminogen-Glu^311^—clinical and therapeutic implications

The data presented suggest Plm-Glu^311^ causes angioedema by a mechanism with features that distinguish it from HAE due to C1-INH deficiency or FXII-Lys/Arg^309^. In brief, the Glu^311^ substitution converts plasmin into a kininogenase that is more efficient than Plg-Lys^311^, with the capacity to release bradykinin at comparable rates from HK and LK. Recently, Hintze et al. confirmed that Plm-Glu^311^ releases bradykinin from HK more rapidly than Plm-Lys^311^ (Hintze et al., 2023). The higher plasma concentration of LK suggests it, and not HK, is the major kinin source during angioedema in carriers of Plg-Glu^311^. The KKS does not appear to be necessary for bradykinin generation in this disorder. These features may provide insight into some interesting clinical observations.

The dependence of the proposed mechanism on fibrinolysis suggests that bradykinin production would be greatest in tissues where fibrinolysis is most active. There is a predilection for oral-lingual angioedema in Plg-Glu^311^ carriers (Bork et al., 2018; Bork et al., 2020a). Tongue swelling occurs in 80% of symptomatic patients, and this is often the only manifestation of HAE (Bork et al., 2018; Bork et al., 2020a; Bork et al., 2020b). Edema of the larynx, extremities, or GI tract is less common, and erythema marginatum, a prodromal rash associated with C1-INH deficiency, is rare in Plg-Glu^311^ carriers. Observations of patients with bleeding disorders suggest that intrinsic fibrinolytic activity is higher in the oral cavity than in most other tissues (Gailani et al., 2023). The predilection for oral-lingual edema in Plg-Glu^311^ patients may, therefore, reflect the normally brisk plasminogen activation in the mouth. Angiotensin-converting enzyme (ACE) inhibitors inhibit bradykinin degradation and can trigger oral-lingual angioedema in some patients (Carucci et al., 2020; Wilkerson and Winters, 2022). Fibrinolytic inhibitors such as tranexamic acid have been effective in treating ACE-inhibitor induced angioedema (Beauchêne et al., 2018), while recent studies suggest that C1-INH infusion is no better than placebo in this situation (Perza et al., 2020; Strassen et al., 2022). Taken as a whole, these observations suggest that basal bradykinin generation is normally greater in the oropharynx than in other tissues, and that plasmin may contribute significantly to bradykinin formation in this area of the body.

Clinical observations, and the work presented here, also raise the possibility that some of the therapeutic options commonly used to treat HAE patients with low C1-INH, including C1-INH infusion and PKa inhibition, may be less effective in patients with Plg-Glu^311^ than in other forms of HAE. Consistent with this notion, Bork and colleagues reported that the mean duration of angioedema episodes in patients with Plg-Glu^311^ was substantially longer after C1-INH infusion (mean duration decrease from 48.2 +/−32.5 h untreated to 31.5 +/−8.6 h treated) than with the B2 receptor antagonist icatibant (mean duration decrease from 44.7+/−28.6 h untreated compared with to 4.3 +/−2.6 h treated) (Bork et al., 2020b). While investigations into optimal treatments for HAE based on underlying mutations are at an early phase, available mechanistic and clinical data suggest that strategies targeting the KKS will not be satisfactory for all patient types.
